# Ten Steps to Design a Counseling App to Reduce the Psychosocial Impact of Human Papillomavirus Testing on the Basis of a User-Centered Design Approach in a Low- and Middle-Income Setting

**DOI:** 10.1200/GO.22.00168

**Published:** 2022-10-17

**Authors:** Victoria Sánchez Antelo, Lucila Szwarc, Anabella Le Pera, Paula Fredjkes, Diana Saimovici, Silvia Massaccesi, Melisa Paolino, Kasisomayajula Viswanath, Silvina Arrossi

**Affiliations:** 1 Consejo Nacional de Investigaciones Científicas y Técnicas, Centro de Estudios de Estado y Sociedad, Buenos Aires, Argentina; 2 Centro de Estudios de Estado y Sociedad, Buenos Aires, Argentina; 3 Instituto Provincial del Cáncer, Ministerio de Salud de la Provincia de Buenos Aires, Buenos Aires, Argentina; 4 Department of Social and Behavioral Sciences, Harvard T.H. Chan School of Public Health, Boston, MA; 5 Dana-Farber Cancer Institute, Harvard University, Boston, MA

## Abstract

This study describes the 10 steps followed to produce the information architecture of a user-centered design (UCD) counseling mobile application, the first phase to develop an app. The app aims to reduce the psychosocial impact of the human papillomavirus test result and improve women's knowledge of human papilloma virus and cervical cancer.

**METHODS:**

We used a UCD approach to produce the information architecture of the app (ie, how to organize contents into features). We analyzed field notes, meeting agendas, and documentation produced during each stage of the design process. We described the goals, methods, and outcomes of each step. We also discussed the critical challenges and the strategies to address them.

**RESULTS:**

The steps are (1) knowledge, attitudes, and beliefs mapping: reanalysis of team's research findings from prior studies; (2) environmental scanning of apps available on the market; (3) stakeholders' point of view: The International Advisory Committee; (4) potential user's profile: building archetypes through the Persona method; (5) women's interviews: user's preferences and experiences; (6) effective features: scoping review to select app's features that address psychosocial impact; (7) the user journey: ideal interaction with the gynecological service and the counseling app; (8) women's focus groups: using Personas and Scenarios to discuss app's mock-up; (9) women's design sessions: prototype test and card-sorting techniques; and (10) team's design session: translating results into visual objects and features.

**CONCLUSION:**

We provide here detailed descriptions of the UCD process of an app for human papillomavirus–tested women for those venturing into the area of mHealth strategies work. Our experience can be used as a guide for future mHealth app development for a low- and middle-income setting.

## INTRODUCTION

Cervical cancer (CC) mortality is a major public health issue. In Argentina, every year, 4,500 new cases are diagnosed, and 2,300 women die from this disease annually.^1^ High CC mortality is related to problems in the cancer control continuum, including lack of follow-up, diagnosis, and treatment.^2,3^ It is expected that human papillomavirus (HPV) vaccine programs and HPV testing will lead to the elimination of CC over the next decades.^4^

**CONTEXT**

**Key Objective**
What steps were followed to produce the information architecture of a user-centered designed app aimed to human papillomavirus (HPV)–tested women?
**Knowledge Generated**
We followed 10 steps to produce the information architecture of a counseling app for HPV-tested women designed with a user-centered approach. For women, the app should mainly provide information on HPV, cervical cancer, and steps to follow after different HPV test results. Information provision turned out to be the organizing axis of the design, and it became a prominent element in the different screens. The app should be mainly offered by health providers, include appointment reminders for follow-up and treatment, and present stories and testimonies of HPV-positive women.
**Relevance**
The study results are key for the implementation of a mHealth-based intervention aimed at providing counseling and increasing women adherence to follow-up/treatment in low/middle-income settings, with high potential contribution to the achievement of WHO targets to eliminate cervical cancer.

As HPV testing detects a sexually transmitted infection with oncogenic types of HPV, HPV positivity can have connotations of promiscuity and stigmatization and can result in shame, worries about future sexual relationships, and questions about the women's or her partner's sexual behavior. HPV positivity can also produce anxiety, fear of cancer or death, and disease denial.^5-7^ The HPV psychosocial impact might not only diminish women's quality of life but also reduce their ability to complete diagnosis and treatment.^8^

Providing information that meets women's needs and counseling are key in alleviating HPV-related distress and its possible impact on adherence to follow-up.^9,10^ The WHO recommends counseling as a strategy for interpersonal communication between health providers and women, allowing them to become more informed and knowledgeable about HPV and CC prevention, to discuss sensitive topics (eg, sexuality), and encourage them to adopt preventive practices.^11^

Although face-to-face counseling would help women overcome the psychosocial impact, its implementation presents many difficulties such as lack of trained providers,^12^ lack of sufficient time and privacy during consultations, lack of awareness about the importance of counseling, and embarrassment when raising issues concerning sexual behaviors.^13^ As a result, HPV-tested women often receive insufficient and/or confusing information about the use and benefits of HPV testing and how to follow-up.^14^

We need innovative and scalable solutions to enhance provision of information, counseling, and support to HPV-tested women that will allow a more efficient use of human resources and time and improve women's autonomy in accessing patient-centered information. mHealth interventions can enhance the relationship between patients and health providers^15-17^; in particular, the use of mobile applications (apps) to communicate with patients and provide support has been shown to improve health outcomes for different health conditions.^18-20^ Apps can be used even after the consultation ended and require less staff.^17,18^ In cancer care, apps offer the possibility of providing accessible information and education at minimal costs throughout the cancer care continuum.^20^

Nonetheless, the literature has shown that most downloaded apps were used only once because users did not immediately engage with them, undermining the intervention's potential effectiveness.^21,22^ Additionally, people's intention to use apps for self-care purposes is influenced by perceived usefulness and ease of use, performance expectancy, social influence, self-efficacy, and potential lack of privacy. Evidence shows that involving end-users in the app design may enhance its perceived utility and usage.^23,24^ In addition, the app proposed features should be integrated within a theory-driven health intervention oriented to behavior change.^25^

We propose an app for HPV-tested women designed with a user-centered approach, whose included features will be integrated within a theory-driven health intervention. The app will provide information and support to HPV-tested women from HPV testing and results delivery to treatment if needed. The app would constitute a low-cost, easy-to-use tool that would change how HPV-tested women access evidence-based information and counseling.

The first phase of an app design is producing the information architecture (IA) which can be summarized as the organization of contents and objects (eg, screens and features), the clear descriptions of their functions and interactions, and the provision of ways for the user to get to them (eg, understandable labels and navigation flows).^26^ The IA is a critical dimension of an app design as it will clearly state its format, presentation, and functions which are key elements for intuitive access to app content and task completion.^27^

This study describes the 10 steps followed to produce the IA of a user-centered design (UCD) counseling app for HPV-tested women. The description dwells on the contribution of each step to the design, the techniques used, and the findings obtained. The study results are key for the implementation of a mHealth-based intervention aimed at reducing the psychosocial impact of HPV testing and increasing women adherence to follow-up/treatment, with high potential contribution to the achievement of WHO targets to eliminate CC.^4^

## METHODS

### Framework

**FIG 1:**
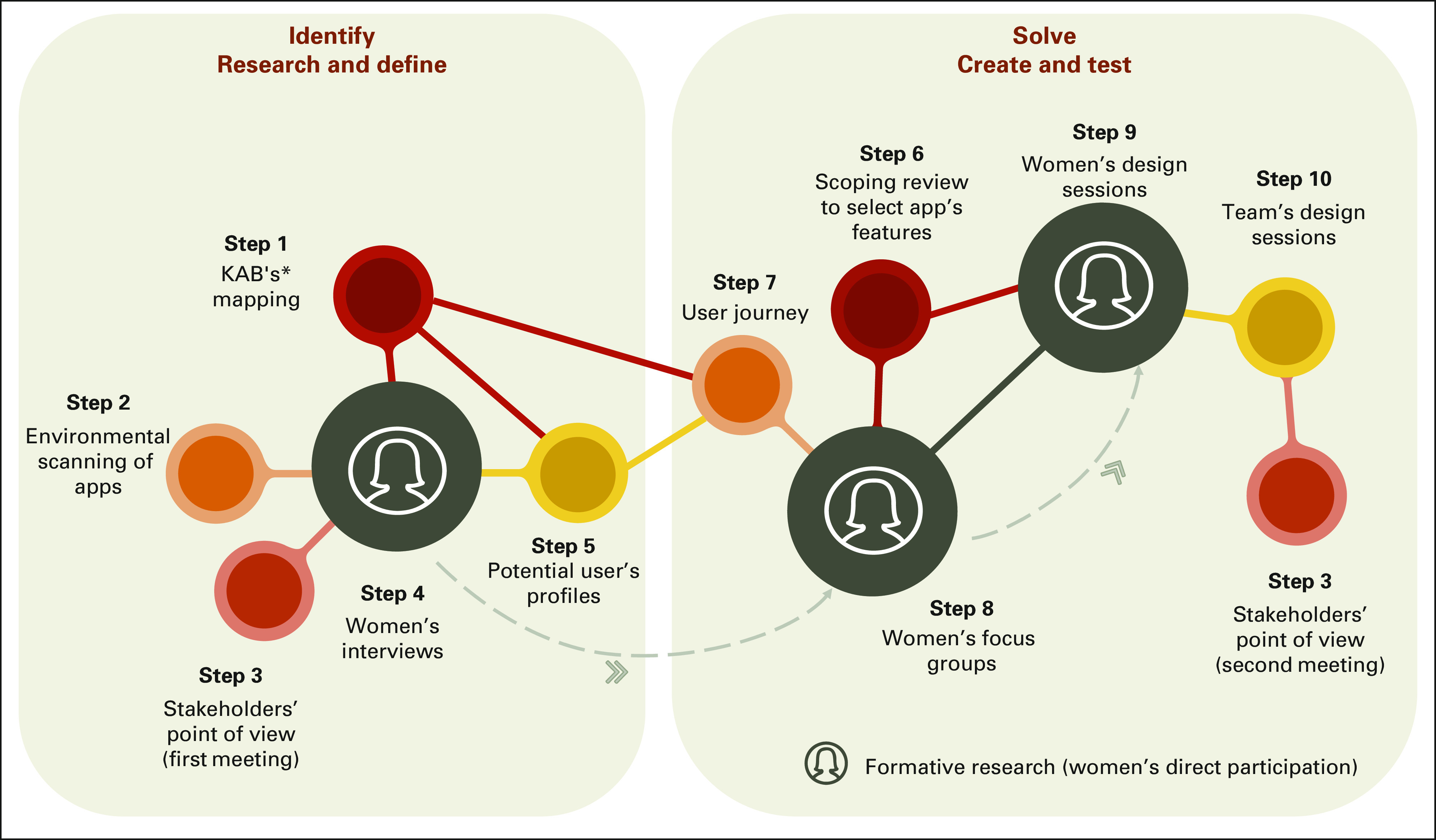
Design-thinking phases and the 10 steps to produce the app's information architecture. *KABs mapping toward HPV testing and follow-up procedures. HPV, human papillomavirus; KABs, knowledge, attitudes, and beliefs.

We relied on the design thinking framework, which is a user-centered framework which provides a guide to translate the intervention aims into technological components and specifications for digital behavioral change interventions.^28^ In addition, the process of designing the IA incorporated a series of different methods and participatory techniques which were iteratively applied^23^ through two major phases: identifying problems (through researching and defining) and solving problems (by creating and testing). Figure 1 shows how the 10 steps described in this study are integrated into the two phases of the IA design.

Formative research was a main activity of the IA design process (extensively described elsewhere).^29^ We conducted online individual interviews (step 4), focus groups (FGs) (step 8), and design sessions (DSs) (step 9) with HPV-positive women age 30 years or older aimed at understanding their perceptions about the app and to investigating their preferences regarding the app design, content, and framing. All other steps shown in Figure 1 were used as additional inputs to the app's iterative design.

### Formative Research Setting

The study took place in Ituzaingó, a district located in the Metropolitan Area of Buenos Aires. In 2015, HPV testing was established as primary screening for women age 30 years or older attending the public health system, mainly the population not covered by the social security sector (such as workers in the informal economy and their families). The public health sector comprises a network of primary health care (PHC) units linked with the secondary level of care that provide care free of cost, including screening, diagnosis, and treatment if needed.

According to Argentinean recommendations,^30^ HPV-positive women are triaged with cytology. Women with abnormal Paps are referred to colposcopy and biopsy if needed. Women with histologically confirmed CIN2 or worse are referred for treatment. HPV-negative women are recommended rescreening in 5 years. HPV-positive women with normal cytology are recommended rescreening in 18 months.

### Participants of Formative Research

Eligible women were literate, age 30 years or older, residing in Ituzaingó, and mobile phone users. We used a purposive sampling procedure among women HPV tested in the past 12 months at Ituzaingó public health institutions. We conducted fieldwork during the COVID-19 pandemic (November-December 2020 and August 2021). Despite this unprecedented context, we recruited 29 women for individual interviews, of whom 19 (66%) participated in the four FGs and 11 took part in the two DSs.

The formative research study's protocol was approved by the Diagnóstico por Imagen Morón private clinic's ethics committee. Women provided verbal informed consent, which was audio recorded for documentation. Anonymity of participants was guaranteed.

### Data Collection and Analysis Used Through the 10 Steps of the App Design Process

We analyzed results from formative research, field notes, documentation produced during each step (such as memos and visual products), and publications of partial results.

For each step, we described its goals, techniques, and outcomes. Thematic analysis^31^ was used to analyze data described in steps 1, 5, and 8 (Fig 1), relying on the Health Belief Model and the Integrative Behavioral Model frameworks, which has been extensively described elsewhere.^29^ The analysis allowed us to organize data into themes, detect emerging topics, and get richer data.

## RESULTS

### Step 1—KABs Mapping

Sep 1 was aimed at systematizing patterns in women's knowledge/attitudes/beliefs (KABs mapping) toward HPV testing and follow-up. For that, we reanalyzed qualitative data obtained in previous studies by our research team, which allowed us to make reinterpretations considering new research questions.^32^

We examined three different corpora of data: first, from interviews carried out in 2012 among women age 18-55 years in the province of Buenos Aires who had abnormal Pap smear results (n = 30)^33,34^; second, from interviews (n = 38) conducted among HPV-tested women in 2013 in the province of Jujuy^7,14^; and finally, data from six FGs carried out in Jujuy with women age 30 years or older (n = 48) who had performed HPV self-collection in 2016-2017.^35^

On the basis of Health Belief Model and Integrative Behavioral Model, we classified data into the following themes: knowledge, attitudes, beliefs, perceived barriers, perceived self-efficacy, and cues to adhere to follow-up. Then, we distinguished emergent subthemes and selected the quotes that better described each theme (Table 1). The KABs mapping was a main input for step 5 (end-users’ profiles), step 7 (user journey), and steps 4 and 8 (interview and FG guides, respectively).

**TABLE 1:**
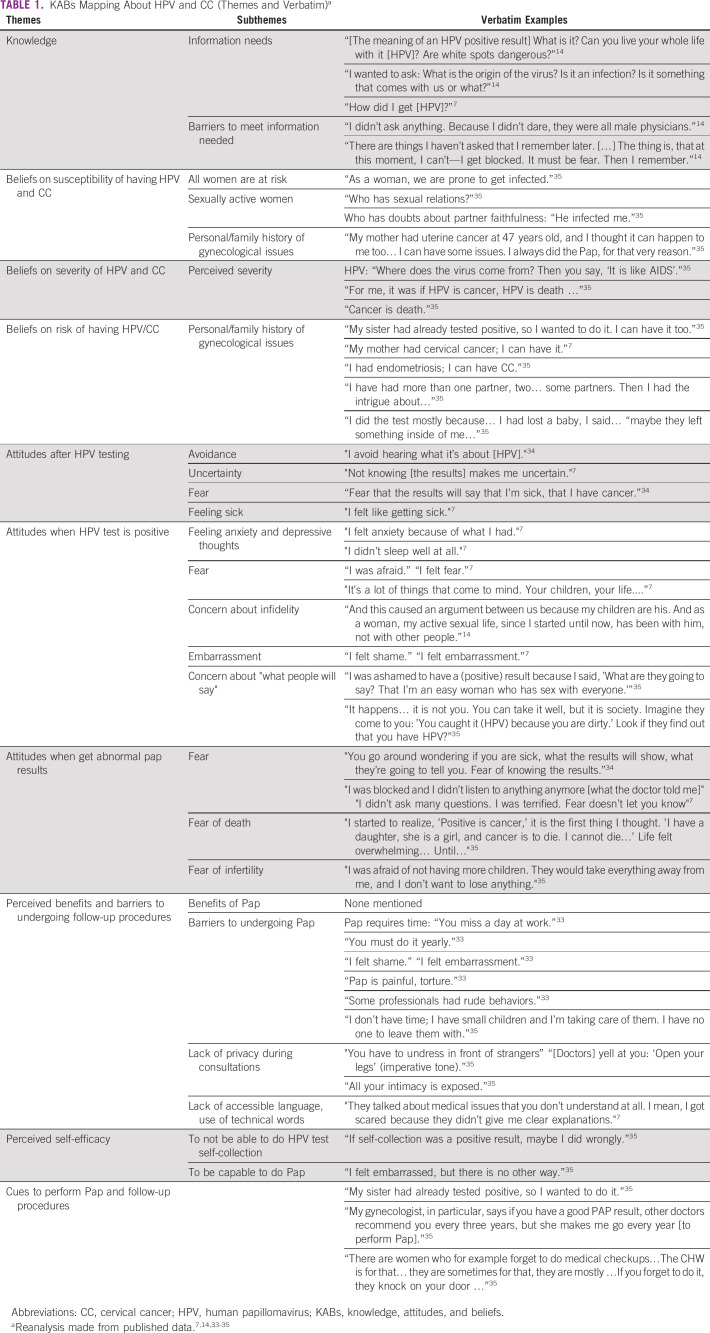
KABs Mapping About HPV and CC (Themes and Verbatim)^a^

### Step 2—Environmental Scanning of Apps

We conducted an environmental scan to explore and characterize the current landscape of apps related to HPV and CC. Between June 1, 2020, and June 14, 2020, we searched both the Apple and Google app stores using the key terms HPV and CC both in English and Spanish. Search update was done in December 2021. Apps oriented at health professionals and those focused on general aspects of gynecology and/or sexuality were excluded.

We summarized the following variables: app name; developer, owner, and supporting institution; target audience; main features; availability (free or paid); year of release; main topic repository category; languages; and operating systems (iOS, android, or both).

We found 19 apps published between 2014 and 2021. All of them were in English while some were in other language (Turkish, Spanish, Italian, or Portuguese), with English as second option. Nearly half of the apps fell into the health/fitness category, followed by medical. Most apps were free and aimed at everyone. Only apps related to HPV vaccination were targeted to a specific audience (mostly parents and very few to teens). The most frequent topics were information about both HPV vaccination and CC prevention. The app main features were to provide information through texts or infographics, remind appointments, and visualize user progress (eg, remaining vaccination doses). All apps were available in both operating systems.

### Step 3—Stakeholders' Point of View: The International Advisory Committee

We established an Advisory Committee of national and international stakeholders, including:

 • the Chief of the Unit of Non-communicable Diseases, Violence, and Injuries Prevention Pan-American Health Organization from the WHO;
 • the Head of Prevention and Implementation Group at the International Agency for Research on Cancer from the WHO;
 • the Head of Oncogenic Viruses Laboratory HPV National and Pan-American Health Organization Regional Reference Laboratory for the WHO HPV-LabNet National Institute of Infectious Diseases-ANLIS “Dr Malbrán”;
 • the President of the Argentinean Society of Lower Genital Tract and Colposcopy;
 • the Head of the National Program on Cervical Cancer Prevention, National Cancer Institute (Argentina);
 • the Coordinator of the Working Group on Communication and Health, “Gino Germani” Institute, University of Buenos Aires; and
 • a renowned female gynecologist and influencer with high expertise in social media.

Key comments of the Advisory Committee were the need to include in the app's implementation a training plan for PHC providers and the importance of considering potential confidentiality issues and data protection regulations not only in Argentina but also in other countries. They also emphasized the importance of the app as a tool to enhance women-health professionals' relationships and to provide clear and evidence-based information to women. Additionally, they highlighted the app as an opportunity to contribute to evidence dissemination not only among women but also among health professionals.

### Step 4—Women's Interviews

Twenty-nine individual online semistructured interviews were conducted among HPV-tested women (see *Participants*). We collected information about use of technology (use of mobile phones, Internet; health apps, preferences regarding apps' features) and women's personal experiences during HPV testing and result delivery.

The results showed that women used their mobile phones daily. Most mentioned the use of apps to record their menstrual cycle, and some used apps to improve their physical activity. Although most women preferred to get first-hand information from doctors, almost all googled health information. Most women felt that they lacked time during the medical consultation to ask questions about HPV positivity and to clarify their doubts. Many felt overwhelmed by the HPV test results and were not able to understand the information provided by the physician. Others did not get much information from the provider. We used these findings to fit the Persona and their scenarios (step 5).

### Step 5—Potential User's Profiles

Five archetypical profiles of HPV-tested women who might be potential app end-users were identified. We used the Persona method,^36^ which is a description of fictitious users on the basis of the categorization of relevant information about real people to build typologies. It was based on the KABs mapping data (step 1) and results of the individual interviews with HPV-tested women (step 4).

We built five Personas on the basis of the following dimensions: sociodemographics, personal interests, partnership situation, common attitudes and behaviors related to health, information needs on HPV testing and CC prevention, structural factors that might deter access to health care (child-caring, geographical distance, etc),^33^ previous experiences that could shape women-provider interaction,^35^ use of technology, and likelihood to adhere to follow-up.^14^

**FIG 2:**
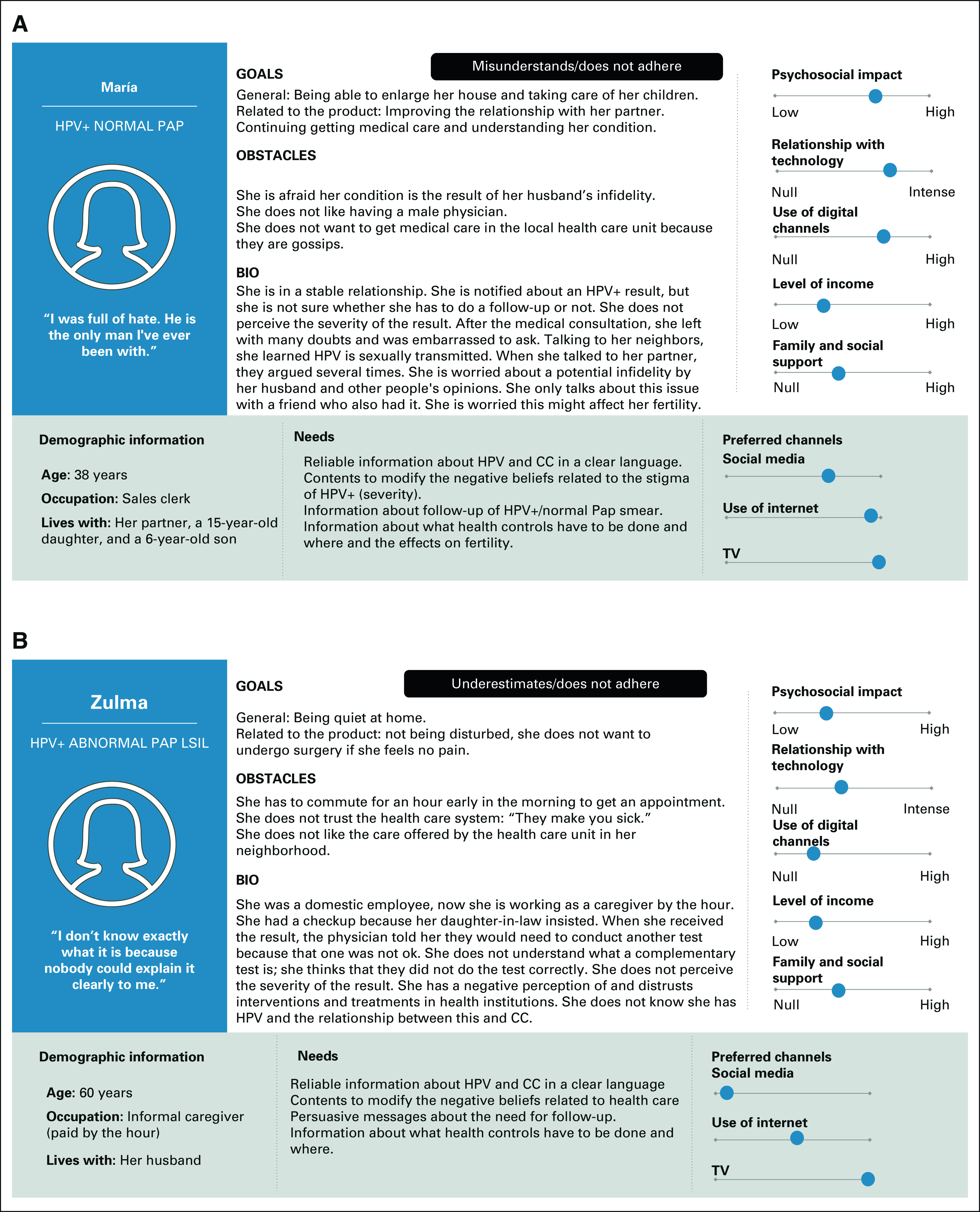
(A and B) Illustrative examples of end-users built using the Persona method. CC, cervical cancer; HPV, human papillomavirus.

We used Personas as potential app users main characters of different scenarios (Fig 2), presented as short stories that described a context where someone might use the app.^26,36^ App components were created as a tool to respond to these Persona/scenario needs (steps 7, 8, 9, and 10).^36^

### Step 6—Scoping Review to Select App's Features

We conducted a scoping review to identifying app-based interventions evaluated in randomized controlled trials with demonstrated efficacy in changing the psychosocial impact of health problems. This review allowed us to map apps with demonstrated effectiveness in influencing intention toward behavior change. We used this list to discuss the app's design during FGs (step 8).

We searched in PubMed, Cochrane, Scopus, SciELO, Embase, British Nursing Index, and PsycInfo databases, where 258 articles were initially found. After eliminating duplicates and applying inclusion criteria, 59 articles were selected and systematized using the PICO format (population, intervention, control group, and outcomes).

We identified 21 interventions using apps reported as effective for KABs and/or behavior change. They offered the following features: sliding cards with texts, audios, videos, or infographics to provide information; chat rooms/forums with health counselors and/or other patients; learning video games (referred to as serious games); links to websites; texts listing things that please the user (eg, photos of loved ones); recording activities or setting tasks with a goal of improvement; reminders; a function to store health data; and a function to provide information on nearby health centers and the name of the attending physician.

### Step 7—The User Journey

We created a roadmap visualizing the ideal user-gynecological service app interaction. We used a technique called User's Journey Map^26^ to display all potential contact points with health providers during HPV testing, results delivery, and follow-up from the end-user point of view. We identified when the app should be offered and used by women.

**FIG 3:**
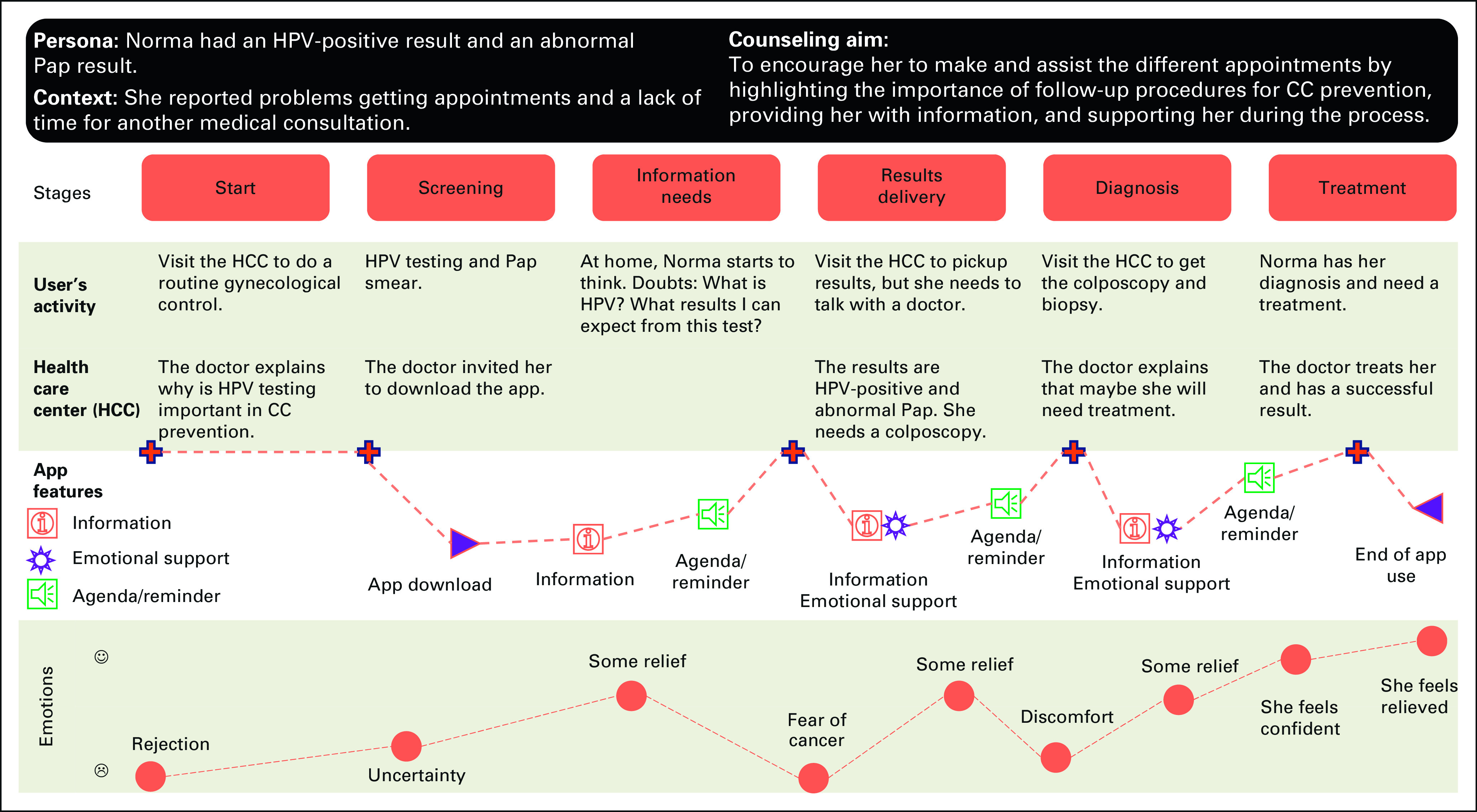
Ideal journey map to HPV-positive and abnormal Pap smear result women. CC, cervical cancer; HPV, human papillomavirus.

We produced scripts of an ideal woman gynecological service interaction on the basis of the KABs mapping (step 1) and Personas (step 5). We debated how counseling by a health provider might be translated into the app's features. As a result, we displayed the ideal user journey of a fictitious woman while using the app (Fig 3).

### Step 8—Women's Focus Groups

Nineteen HPV-positive women participated of the online FGs. Women's age was the segmentation criterion: two FGs included women age 30-49 years and two included women age 50 years or older.

FGs included two participatory activities. First, participants were asked to comment on two different Personas and scenarios that presented two different HPV results delivery situations.^26,36^ On the basis of the steps 4, 5, and 7, scenarios depicted an encounter between a woman and the health care system for HPV testing, cytology triage, and results delivery. We asked participants the following questions: After receiving the results, how did this woman feel? What did she do next? Did/would she use an app to cope? If yes, what characteristics should the app have to be useful?

A second activity, described elsewhere,^29^ was aimed at understanding women's views on an app's model called mock-up. The mock-up consisted of seven screens that included labels of the content, menus of options, and draft illustrations. This allowed us to show women understandable visual triggers to debate the concepts presented on each screen. We asked them about their preferences for app features and screen organization.

The results of FGs indicated that women accepted the app as a tool to obtain information and reduce fears related to HPV-positive results. For them, the app should primarily provide information about HPV and CC, over other functions such as coping strategies that we suggested. The women stressed the importance of the app conveying an empathetic communication style (“as if we were being listened to by an empathetic counselor, with a warm attitude”). They suggested that the app could facilitate connections between women going through a similar process. Finally, women asked for the inclusion of a reminder function of medical appointments and to pick up results.

### Step 9—Women's Design Sessions

A DS has a FG format where participants provide feedback regarding app prototypes, contribute to design ideas, and explain their preferences regarding app functions and tasks.^23^ We conducted two DS with women who had participated in the interviews and FGs (steps 4 and 8): one DS included women age 30-49 years, and the other included women age 50 years or older.

First, we used a card-sorting technique to understand how participants organize and label concepts^37^ and classified topics that fell under information needs. On the basis of women's common doubts or concerns identified in steps 1, 7, and 8, we built cards with different questions about HPV. We showed participants these cards and a list of categories that simulated the labels of the application's menus. Then, women were asked to match each card with a label, ie, with which of the labels they associated the content of the card. Women shared and justified their choices and criteria used in their classification. Finally, the session moderator summarized the group's agreements. The results were used to select the final labels included in the IA. A main finding of this activity was that, to obtain information and resolve doubts, women preferred to use the questions and answers and tests and controls categories.

Second, we used a low-fidelity clickable prototype using a PowerPoint presentation to assess two critical elements: (1) comprehension of the Agenda section (functionality, navigation, and wording) and (2) women acceptability to use a log in username and password to protect confidentiality. We presented an interactive prototype, and we invited women to simulate the setting of a reminder to pick up the HPV test result. When the required fields were completed, the prototype app provided them with information about the next steps. During the process, we used open-ended questions to assess comprehension and preference elicitation.

The results from the prototype assessment showed that women accepted the Agenda features to obtain synchronized information. They also considered that the sequence of the proposed screens was easy to understand and navigate. Additionally, they highlighted the importance of including a feature to store data and/or files related to their gynecological consultations and HPV test results. Some suggested displaying a graph visualizing their progress in treatment (ie, woman's progress along the continuum of care).

Regarding confidentiality issues, women described it as annoying to use a username and password to log in and retrieve clinical information. Nevertheless, they agreed on the importance of preserving their data and acknowledged that they were familiar with this type of log in and passwords functions.

### Step 10—Team's Design Sessions

**FIG 4:**
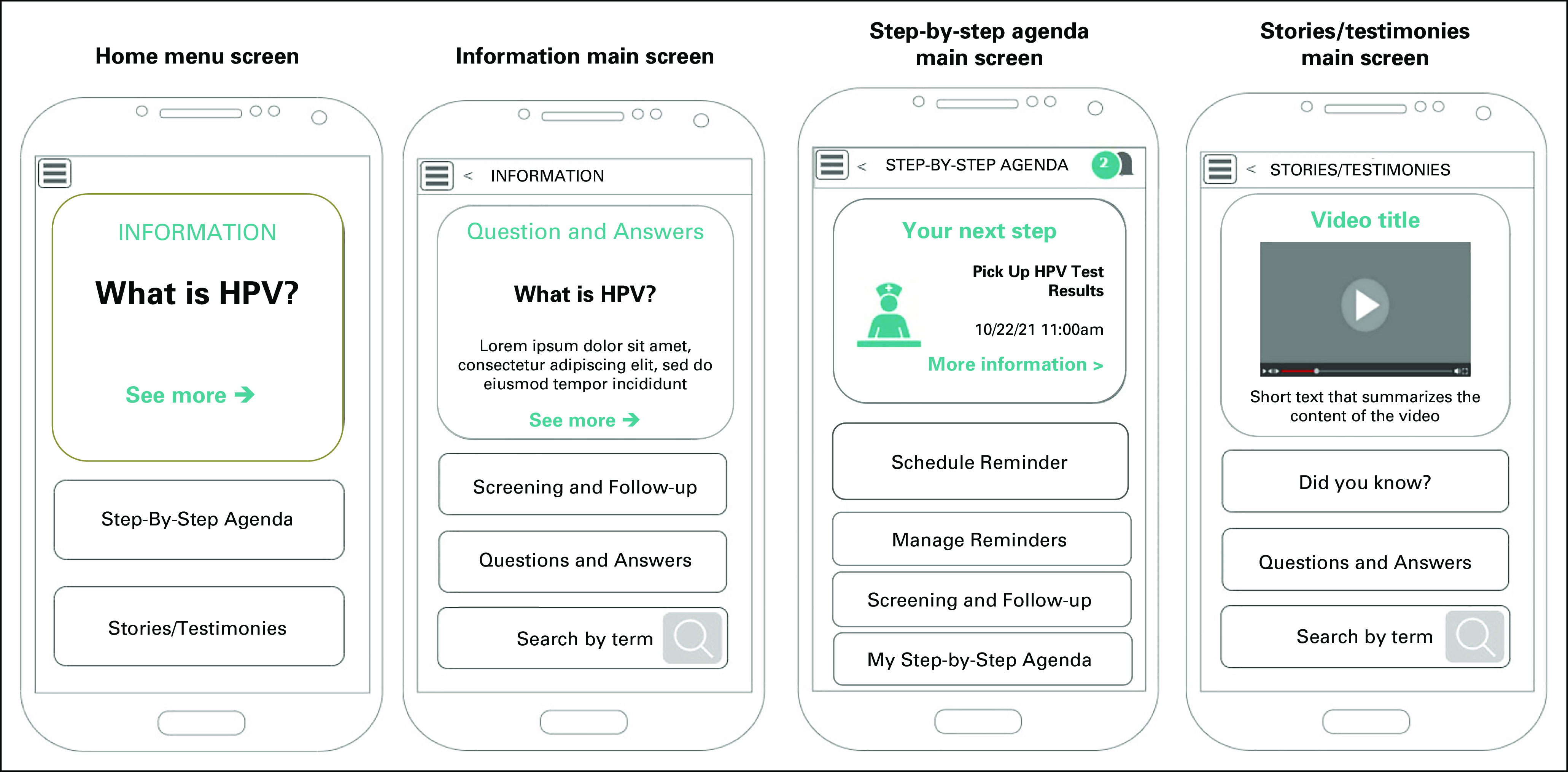
Paper prototypes of main screens. HPV, human papillomavirus.

The team DS was aimed at translating results of previous steps into a set of app's screens with detailed features (Fig 4). The project team included five sociologists, two psychologists, a user-experience/graphical design expert, a philosopher, and a gynecologist who works in PHC. We used the Crazy 8^38^ technique, an exercise suitable for multidisciplinary groups where participants have 8 minutes to sketch the app screens and provide innovative solutions. The main results of this activity were as follows:

The app's main objective should be to provide information on HPV and CC. This objective turned out to be the organizing axis of the design, and it became a prominent element in the different screens. A visual hierarchy of the informational contents was developed by the team. Thus, the main menu (Home Menu) was designed to contain a clickable box with highlights from the HPV Information section that is visually bigger than other buttons.

Women's demand to be heard by an empathic counselor was translated into the app's different design components. Thus, illustrations and wording of the screens emulated an empathic dialogue of a fictitious health professional with the user. We also included a feature where women could type their doubts using keywords to get specific information (feature Search by term and Step-by-step agenda).

Women’s demand to connect with other HPV-positive women was translated into a section called Stories and testimonies where they would be able to watch stories on the basis of real stories, narrated in the first person and a feature to share the content of the app with other women.

### Final Information Architecture

**FIG 5:**
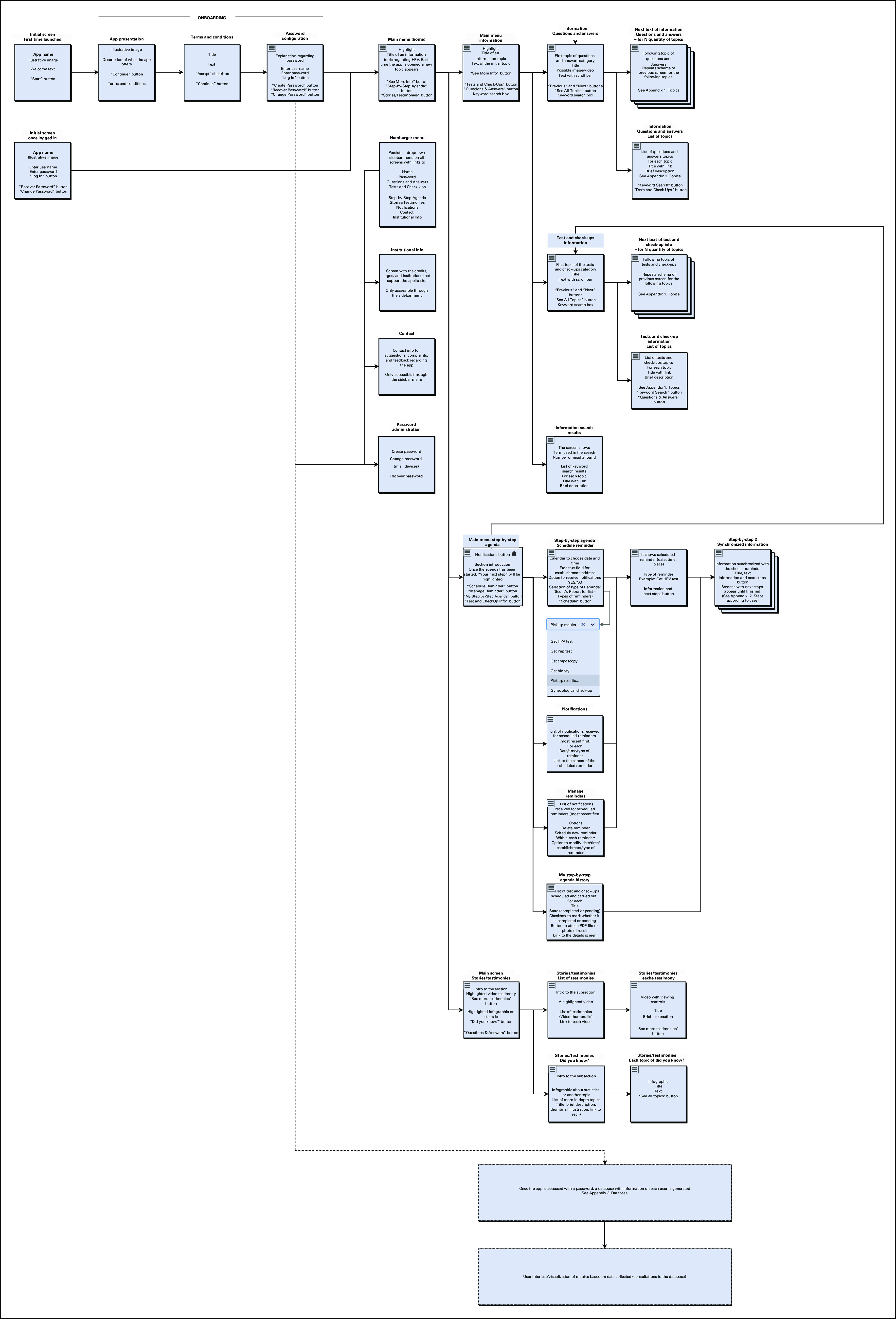
Information architecture (screens and features) map.

The final IA (Fig 5) presents the screen sequence and content organized into three sections: Information, Step-by-step Agenda, and Stories and testimonies. The information section will provide medical/scientific information about HPV and CC prevention, explained in clear and accessible language. It is composed of two subcategories: Questions and Answers; and Screening and Follow-up. The Step-by-step Agenda section will provide women with synchronized information regarding medical appointments. Finally, the Stories and testimonies section will include the stories of HPV-positive women regarding HPV and CC in clear language.

## DISCUSSION

In this study, we described the 10 steps to produce the IA of an app aimed at providing information, reducing the psychosocial impact of HPV testing, and counseling HPV-tested women. We used a UCD approach that implied identifying the users' needs, design preferences, and the facilitators for uptake and sustained use of the app. For an app aimed at a behavior change intervention, the design needed a balance between the users' preferences and an evidence-based and theory-driven design. We relied in design thinking framework to face this challenge, and we described how we translated it into a coherent IA.

The app design was based on formative research conducted among urban women living in the Metropolitan Area of Buenos Aires. The results showed that women reported unmet information needs regarding HPV and follow-up steps^29^ and that is why for them the app's main objective should be to provide information on HPV, CC, and steps to follow after different HPV test results. This turned out to be the organizing axis of the design, and it became a prominent element in the different screens.^29^ Studies carried out in several rural and urban settings from Argentina^7,14,33^ and Latin America and among Hispanic women from the United States^39-41^ found similar information needs. Therefore, our IA on the basis of the information needs and psychosocial concerns of urban women from Argentina might be also usable in other regional contexts, whether rural or urban. Clearly, some app adaptations will be needed regarding specific aspects highly influenced by the sociocultural and health system environment.^42^ Thus, in certain settings, it might be necessary to test the wording to adjust the language to the variations of Spanish within LA or translate it into Portuguese for the Brazilian audience. In addition, diagnosis/treatment algorithms might vary, so specific adaptations will be needed to provide women with information about how to continue the line of care. Thus, the app IA has provided a design map about how content should be organized and navigated, but the specific, locally dependent information that will be included could be adapted to different settings.

mHealth interventions are particularly suited to assist underserved people accessing health services irrespective of time, place, and person.^43^ Previous research^44^ has shown that mHealth-based interventions can be more effective among rural women as they constitute a powerful tool to connect rural women with the health system. Thus, the app might be an intervention particularly suitable to increase rural women access to HPV-related information and resources. Some concern exists regarding lower mobile phone use in rural areas,^45^ which might hinder use of the app in those settings. At present, around 73% of the Latin American population has a mobile phone,^46^ and in Argentina, this figure is 84%.^47^ Additionally, the mobile phone penetration rate is rapidly increasing in the region, especially after the COVID-19 pandemics.^46^ Therefore, it is possible to hypothesize that even if at earlier stages of the app implementation some rural women might not be reached, on the long-term, as the urban-digital divides closes, the app might be used equally implemented both in rural and urban settings.

Dissemination refers to how evidence-based practices are communicated among potential adopters and implementers to produce uptake and effective use.^48^ Our study showed that women would use the app specially if recommended by health professionals and endorsed by health authorities. Thus, we envisaged the app as a tool to be offered by health professionals to women during screening-related consultations, enhancing provider-patient communication. Research has shown that early involvement of implementers in intervention design is a facilitator of its adoption.^49,50^ In our study, both potential adopters and decision makers views were included in the IA design phase mainly through its inclusion in the Advisory Committee, where they commented and provided advice on the design process. Very importantly, they were favorable to the app design and implementation and considered that it would fill a gap in the screening process. There was also agreement in visualizing the implementation of the app as potentially integrated into the set of strategies included in municipal/provincial/national screening policies. In addition, we are at present carrying out formative research to incorporate health professionals' views about obstacles and facilitators of the app implementation/dissemination. This will allow design of strategies to address contextual barriers and guarantee successful adoption of an app-based intervention.^51,52^

A main barrier to incorporating an app as part of health services provision is a potential low rate of app download/use. To reduce this problem, we used a UCD approach, as it allows considering needs of users, app design preferences, and barriers and facilitators that prohibit or encourage app uptake and sustained use.^27^ Increasing the app download/use can also be achieved through appropriate implementation strategies. In a study that found that an app-based intervention was effective to increase mammograms among Korean American immigrant women,^53^ study staff downloaded the app into women's mobile phones during the enrollment consultation. The results of that study underscore the importance of developing the app as a tool that should be offered and downloaded during the screening consultation with support by health providers.

Translation of our results into an effective app that is part of a programmatic strategy to prevent CC implies an evaluation of its implementation at different levels. In addition, developing app features and multimedia content involves an iterative process between researchers and potential users to reflect their needs in the app design.^54^ Thus, the evaluation phase will start with usability and quality testing of the app prototype by potential adopters and end-users.^55^ This will include women assessment of the app's content comprehension and interaction experience, with the aim of obtaining a product to be validated in real-life contexts and with larger numbers of women. Finally, evaluation of the strategy will be key to determine if the app implementation is effective to increase women knowledge about HPV testing and decrease the psychosocial impact of HPV positivity. On the basis of Implementation Science frameworks, the evaluation will use quantitative and qualitative methods to assess the app effectiveness, acceptability and adoption, and identify and understand barriers and facilitators of its implementation.

## PREPRINT VERSION

We published the final results from focus groups in the Journal of Medicine Internet Research, Formative Research https://formative.jmir.org/2022/1/e32610.

## References

[1] Bray F, Ferlay J, Soerjomataram I, et al: Global cancer statistics 2018: GLOBOCAN estimates of incidence and mortality worldwide for 36 cancers in 185 countries. CA Cancer J Clin 68:394-424, 2018 30207593 10.3322/caac.21492

[2] Murillo R, Almonte M, Pereira A, et al: Cervical cancer screening programs in Latin America and the Caribbean. Vaccine 26:L37-L48, 2008 18945401 10.1016/j.vaccine.2008.06.013

[3] Arrossi S, Paolino M, Sankaranarayanan R: Challenges faced by cervical cancer prevention programs in developing countries: A situational analysis of program organization in Argentina. Pan Am J Public Health 28:249-257, 2010 10.1590/s1020-49892010001000003 21152712

[4] Das M: WHO launches strategy to accelerate elimination of cervical cancer. Lancet Oncol 22:20-21, 2021 33248466 10.1016/S1470-2045(20)30729-4

[5] Bennett KF, Waller J, Ryan M, et al: The psychosexual impact of testing positive for high-risk cervical human papillomavirus (HPV): A systematic review. Psychooncology 28:1959-1970, 2019 31411787 10.1002/pon.5198 PMC6851776

[6] Arrossi S, Almonte M, Herrero R, et al: Psycho-social impact of positive human papillomavirus testing in Jujuy, Argentina results from the Psycho-Estampa study. Prev Med Rep 18:101070, 2020 32257775 10.1016/j.pmedr.2020.101070 PMC7113430

[7] Szwarc L, Sánchez Antelo V, Paolino M, et al. "Me sentí enfermar": percepciones y comprensión de las mujeres del resultado positivo de un test de virus del papiloma humano en Jujuy, Argentina. Salud Colect 17:e3572, 2021 34752024 10.18294/sc.2021.3572

[8] McLachlan E, Anderson S, Hawkes D, et al: Completing the cervical screening pathway: Factors that facilitate the increase of self-collection uptake among under-screened and never-screened women, an Australian pilot study. Curr Oncol 25:e17-e26, 2018 29507491 10.3747/co.25.3916 PMC5832286

[9] Kahn JA, Slap GB, Bernstein DI, et al: Personal meaning of human papillomavirus and Pap test results in adolescent and young adult women. Health Psychol 26:192-200, 2007 17385971 10.1037/0278-6133.26.2.192

[10] Waller J, McCaffery K, Kitchener H, et al: Women’s experiences of repeated HPV testing in the context of cervical cancer screening: A qualitative study. Psychooncology 16:196-204, 2007 16858719 10.1002/pon.1053

[11] PAHO: Integrating HPV Testing in Cervical Cancer Screening Program: A Manual for Program Managers (ed 1). Washington, DC, Pan American Health Organization, 2016

[12] Evans C, Nalubega S, McLuskey J, et al: The views and experiences of nurses and midwives in the provision and management of provider-initiated HIV testing and counseling: A systematic review of qualitative evidence. JBI Database System Rev Implement Rep 13:130-286, 2015 10.11124/jbisrir-2015-2345 26767819

[13] EngenderHealth: Comprehensive Counseling for Reproductive Health. Trainers’ Manual: An Integrated Approach (ed 1). New York, NY, EngenderHealth, 2003

[14] Szwarc L, Sánchez Antelo V, Paolino M, et al: "I’m neither here, which would be bad, nor there, which would be good": The information needs of HPV+ women. A qualitative study based on in-depth interviews and counselling sessions in Jujuy, Argentina. Sex Reprod Health Matters 29:453-463, 2021 10.1080/26410397.2021.1991101 PMC8604540 34779742

[15] Chen Z, Fang L, Chen L, et al: Comparison of an SMS text messaging and phone reminder to improve attendance at a health promotion center: A randomized controlled trial. J Zhejiang Univ Sci B 9:34-38, 2008 18196610 10.1631/jzus.B071464 PMC2170466

[16] da Costa TM, Salomão PL, Martha AS, et al: The impact of short message service text messages sent as appointment reminders to patients’ cell phones at outpatient clinics in São Paulo, Brazil. Int J Med Inform 79:65-70, 2010 19783204 10.1016/j.ijmedinf.2009.09.001

[17] Kannisto KA, Koivunen MH, Välimäki MA: Use of mobile phone text message reminders in health care services: A narrative literature review. J Med Internet Res 16:e222, 2014 25326646 10.2196/jmir.3442 PMC4211035

[18] Visser A, Prins JB, Jansen L, et al: Group medical consultations (GMCs) and tablet-based online support group sessions in the follow-up of breast cancer: A multicenter randomized controlled trial. Breast 40:181-188, 2018 29906741 10.1016/j.breast.2018.05.012

[19] Graetz I, Anderson JN, McKillop CN, et al: Use of a web-based app to improve postoperative outcomes for patients receiving gynecological oncology care: A randomized controlled feasibility trial. Gynecol Oncol 150:311-317, 2018 29903391 10.1016/j.ygyno.2018.06.007

[20] Jongerius C, Russo S, Mazzocco K, et al: Research-tested mobile apps for breast cancer care: Systematic review. JMIR mHealth uHealth 7:e10930, 2019 30741644 10.2196/10930 PMC6388100

[21] Hall CS, Fottrell E, Wilkinson S, et al: Assessing the impact of mHealth interventions in low- and middle-income countries—What has been shown to work? Glob Health Action 7:1-12, 2014 10.3402/gha.v7.25606 PMC4216389 25361730

[22] Voth M, Chisholm S, Sollid H, et al: Efficacy, effectiveness, and quality of resilience-building mobile health apps for military, veteran, and public safety personnel populations: Scoping literature review and app evaluation. JMIR mHealth uHealth 10:e26453, 2022 35044307 10.2196/26453 PMC8811698

[23] Schnall R, Rojas M, Bakken S, et al: A user-centered model for designing consumer mobile health (mHealth) applications (apps). J Biomed Inform 60:243-251, 2016 26903153 10.1016/j.jbi.2016.02.002 PMC4837063

[24] Kang YN, Shen HN, Lin CY, et al: Does a mobile app improve patients’ knowledge of stroke risk factors and health-related quality of life in patients with stroke? A randomized controlled trial. BMC Med Inform Decis Mak 19:1-9, 2019 31864348 10.1186/s12911-019-1000-z PMC6925878

[25] Bidargaddi N, Musiat P, Winsall M, et al: Efficacy of a web-based guided recommendation service for a curated list of readily available mental health and well-being mobile apps for young people: Randomized controlled trial. J Med Internet Res 19:e141, 2017 28500020 10.2196/jmir.6775 PMC5446666

[26] Spencer D: A Practical Guide to Information Architecture (ed 1). Penarth, Five Simple Steps, 2010

[27] Garrett JJ: The Elements of User Experience: User-Centered Design for the Web and Beyond (ed 2). Berkeley, CA, Pearson Education, 2011

[28] Voorheis P, Zhao A, Kuluski K, et al: Integrating behavioral science and design thinking to develop mobile health interventions: Systematic scoping review. JMIR mHealth uHealth 10:e35799, 2022 35293871 10.2196/35799 PMC8968622

[29] Sanchez Antelo V, Szwarc L, Paolino M, et al: A counseling mobile app to reduce the psychosocial impact of human papillomavirus testing: Formative research using a user-centered design approach in a low-middle-income setting in Argentina. JMIR Form Res 6:e32610, 2022 35023843 10.2196/32610 PMC8796044

[30] Arrossi S, Thouyaret L, Paul L: Prevención del cáncer cervicouterino: recomendaciones para el tamizaje, segui miento y tratamiento de mujeres en el marco de programas de tamizaje basados en el test de VPH. Actualizacion 2015. Buenos Aires, INC-MSAL, 2015

[31] Braun V, Clarke V: Using thematic analysis in psychology. Qual Res Psychol 3:77-101, 2006

[32] Corti L: Re-using archived qualitative data—Where, how, why? Arch Sci 7:37-54, 2007

[33] Paolino M, Arrossi S: Analysis of the reasons for abandoning the follow-up and treatment process in women with pre-cancerous cervical lesions in the province of Jujuy: Implications for health management. Salud Colect 8:247-261, 2012 23681458 10.18294/sc.2012.165

[34] Arrossi S, Ramos S, Straw C, et al: HPV testing: A mixed-method approach to understand why women prefer self-collection in a middle-income country. BMC Public Health 16:832, 2016 27538390 10.1186/s12889-016-3474-2 PMC4990977

[35] Sánchez Antelo V, Kohler RE, Szwarc L, et al: Knowledge and perceptions regarding triage among human papillomavirus–tested women: A qualitative study of perspectives of low-income women in Argentina. Womens Health (Lond) 16:1745506520976011, 2020 33264086 10.1177/1745506520976011 PMC7716054

[36] Nielsen L: Personas – User Focused Design (ed 2). London, UK, Springer‐Verlag, 2019. http://www.springer.com/series/6033

[37] Spencer D: Card Sorting: Designing Usable Categories (ed 1). Brooklyn, NY, Rosenfeld Media, 2009

[38] Kaplan K: Facilitating and Effective Design Studio Workshop. Fremont, CA, Nielsen Norman Group, 2017. www.nngroup.com/articles/facilitating-design-studio-workshop

[39] Liebermann EJ, VanDevanter N, Hammer MJ, et al: Social and cultural barriers to women’s participation in Pap smear screening programs in low- and middle-income Latin American and Caribbean countries: An integrative review. J Transcult Nurs 29:591-602, 2018 29366369 10.1177/1043659618755424

[40] Moore de Peralta A, Holaday B, McDonell JR: Factors affecting Hispanic women’s participation in screening for cervical cancer. J Immigr Minor Health 17:684-695, 2015 24578156 10.1007/s10903-014-9997-7

[41] León-Maldonado L, Wentzell E, Brown B, et al: Perceptions and experiences of human papillomavirus (HPV) infection and testing among low-income Mexican women. PLoS One 11:e0153367, 2016 27149525 10.1371/journal.pone.0153367 PMC4858263

[42] Caplan S, Sosa Lovera A, Veloz Comas E, et al: A mobile app to prevent depression among low-income primary care patients in the Dominican Republic: Sociocultural adaptations. J Transcult Nurs 31:413-424, 2020 32188342 10.1177/1043659620912315

[43] Akter S, Ray P: mHealth – An ultimate platform to serve the unserved. Yearb Med Inform 19:94-100, 2010 20938579

[44] Arrossi S, Paolino M, Sánchez Antelo V, et al: Effectiveness of an mHealth intervention to increase adherence to triage of HPV DNA positive women who have performed self-collection (the ATICA study): A hybrid type I cluster randomised effectiveness-implementation trial. Lancet Reg Health Am 9:100199, 2022 35655914 10.1016/j.lana.2022.100199 PMC9159703

[45] CEPAL: State of Broadband in Latin America and the Caribbean 2017, Santiago, Chile, United Nations, 2018

[46] GSMA: La Economía Móvil en America Latina. Mob Econ 46:1-48, 2021

[47] INDEC: Acceso y uso de tecnologías de la información y la comunicación. EPH Inf Técnicos Cienc y Tecnol 4:1-16, 2020

[48] Dearing JW, Singhal A: New directions for diffusion of innovations research: Dissemination, implementation, and positive deviance. Hum Behav Emerg Technol 2:307-313, 2020

[49] Arrossi S, Paolino M, Laudi R, et al: Changing the paradigm of cervical cancer prevention through introduction of HPV-testing: Evaluation of the implementation process of the Jujuy Demonstration Project in Argentina. Ecancermedicalscience 15:1199, 2021 33889208 10.3332/ecancer.2021.1199 PMC8043686

[50] Alfaro K, Soler M, Maza M, et al: Cervical cancer prevention in El Salvador: Gains to date and challenges for the future. Cancers (Basel) 14:2776, 2022 35681756 10.3390/cancers14112776 PMC9179345

[51] Damschroder LJ, Aron DC, Keith RE, et al: Fostering implementation of health services research findings into practice: A consolidated framework for advancing implementation science. Implement Sci 4:50, 2009 19664226 10.1186/1748-5908-4-50 PMC2736161

[52] King DK, Shoup JA, Raebel MA, et al: Planning for implementation success using RE-AIM and CFIR frameworks: A qualitative study. Front Public Health 8:1-14, 2020 32195217 10.3389/fpubh.2020.00059 PMC7063029

[53] Lee H, Ghebre R, Le C, et al: Mobile phone multilevel and multimedia messaging intervention for breast cancer screening: Pilot randomized controlled trial. JMIR mHealth uHealth 5:e154, 2017 29113961 10.2196/mhealth.7091 PMC5698632

[54] Molina-Recio G, Molina-Luque R, Jiménez-García AM, et al: Proposal for the user-centered design approach for health apps based on successful experiences: Integrative review. JMIR mHealth uHealth 8:1-18, 2020 10.2196/14376 PMC7203616 32319965

[55] Bank C, Cao J: The guide to UX design process & documentation. UXPin 125:83-98, 2015

